# *Myrtus Communis* Liquor Byproduct as a Source of Bioactive Compounds

**DOI:** 10.3390/foods8070237

**Published:** 2019-06-30

**Authors:** Fabio Correddu, Mariateresa Maldini, Roberta Addis, Giacomo Luigi Petretto, Michele Palomba, Gianni Battacone, Giuseppe Pulina, Anna Nudda, Giorgio Pintore

**Affiliations:** 1 Dipartimento di Agraria, Sezione di Scienze Zootecniche, University of Sassari, viale Italia, 39, 07100 Sassari, Italy; 2 Dipartimento di Chimica e Farmacia, University of Sassari, via F. Muroni, 23/b, 07100 Sassari, Italy

**Keywords:** LC-MS/MS, fatty acids, polyphenols, antioxidant activity

## Abstract

The fatty acid (FA), polyphenol content and evaluation of the antioxidant capacity of exhausted *Myrtus communis* berries (EMB) resulting from the production of myrtle liqueur were assessed. All parts of the exhausted berries exhibited high concentrations of carbohydrates, proteins, lipids and phenolic compounds. The lipid fraction contained a high amount of poly unsaturated fatty acids (PUFA), mainly represented by linoleic acid (>70%). Of the phenolic acids evaluated by liquid chromatography/mass spectrometry, ellagic acid was the most predominant (>50%), followed by gallic and quinic acids. Quercetin and quercetin3-O-rhamnoside were the most abundant flavonoids. The seed extracts showed a higher antioxidant potential than the pericarp extracts; the same trend was observed for total phenolic compounds evaluated by spectrophotometric assay. The overall high content of bioactive compounds and the high antioxidant potential of this byproduct sustain its suitability for a number of industrial applications, such as a food ingredient in novel foods, an additive in cosmetic formulations or a component of animal feed formulations.

## 1. Introduction

Over recent years, interest in the recovery of high added-value products from waste plant material has grown worldwide, as the re-use and valorization of these byproducts have become important economic issues in a number of industrial sectors [1].

Food processing waste often consists of organic material, the disposal of which presents a serious pollution risk. However, the appropriate management and disposal of such materials entails additional cost. Attempting to extract extra value out of agricultural waste is thus a major step towards alleviating this problem. Many byproducts arising from the processing of fruit and vegetables are rich in phytochemicals that may still retain valuable chemical and biological properties [2]. For example, it has been repeatedly demonstrated that such byproducts can possess high amounts of phenolic compounds—a large group of secondary metabolites that includes: phenolic acids, flavonoids, anthocyanins, and proanthocyanidins [3]. These metabolites have received a great deal of attention because of their numerous biological properties, such as their anti-mutagenic, cardioprotective, anti-inflammatory, anti-carcinogenic, anti-allergic, antiviral and antioxidant activities [4]. Indeed, myrtle liqueur itself has been reported to exhibit strong antioxidant activity [5]. Furthermore, several epidemiological studies suggest that a diet rich in antioxidants may have a positive impact by increasing the reactive antioxidant potential of an organism and reducing the risk of certain degenerative diseases that originate from deleterious free radical reactions [6].

Polyphenol-rich byproducts could be used as functional antioxidant ingredients in the food industry; this possibility is particularly interesting because the currently available and widely used synthetic antioxidants, such as butylated hydroxyanisole (BHA) and butylated hydroxytoluene (BHT), have been suspected to cause negative health effects [7]. Polyphenol-rich byproducts could also provide attractive solutions for the pharmaceutical and cosmetic industries [8,9].

The utilization of byproducts rich in polyphenols as feedstuff has also been explored since recent studies into ruminants showed beneficial effects on health conditions [10], protein and lipid ruminal metabolism [11,12] and the immune system [13], as well as an enhancement of milk production and quality [14,15]. In addition, their use as feed in animal diets may help avoid expensive byproduct treatments, which often lead to further waste production [1].

To the best of our knowledge, no studies have been performed on the byproducts derived from myrtle liqueur production. The berries of *Myrtus communis* are used to produce a sweet myrtle liqueur by their hydroalcoholic infusion (> 40 °C) lasting at least 15 days [16]. More than three million bottles of myrtle liqueur are currently produced in Sardinia per year, and it is fast becoming one of the most popular Sardinian exports [17]. Of consequence, Sardinia produces a considerable amount of exhausted berries of *Myrtus communis* as a waste product, estimated to be approximately 200,000 tons/year.

The present study aimed to characterize the chemical composition and the phenolic profile of exhausted berries of *Myrtus communis* resulting from myrtle liqueur production and to test their antioxidant activity in order to evaluate their potential for further exploitation.

## 2. Materials and Methods

### 2.1. Reagents and Standards

The solvents used for extraction (methanol, acetonitrile and formic acid) were purchased from Sigma-Aldrich Chemical Company (St Louis, MO, USA). A Milli-Q purification system (Millipore, Bedford, MA, USA) was used to prepare high-performance liquid chromatography (HPLC) grade water. Standards of gallic acid, caffeic acid, p-coumaric acid, ellagic acid, ferulic acid, sinapic acid, quinic acid, syringic acid, chlorogenic acid, phloridzin, kaempferol, luteolin, quercetin, isorhamnetin, myricetin, apigenin, epicatechin and catechin were purchased from Sigma-Aldrich. Standards of quercetin rhamnoside, isoquercetin, rutin, robinin, isorhamnetin rutinoside, neohesperidin, quercetin galactoside, myricitrin, myricetin galactoside, epigallocatechin, epigallocatechin gallate, procyanidin B1, procyanidin B2, cyanidin-3-O-glucoside, cyanidin-3-O-arabinoside, cyanin, delphinidin-3-O-glucoside, malvidin-3-O-glucoside, and pelargonidin-3-O-glucoside chloride were purchased from Extrasynthese (Genay, France).

The reference standard mixture of 37 FAME (FAME mix 37) was acquired from Supelco (Sigma-Aldrich, Bellefonte, PA, USA); other reference standards were purchased from Matreya Inc. (Pleasant Gap, PA, USA): PUFA-2, a nonconjugated 18:2 isomer mixture of individual PUFA, eicosapentaenoic acid, (EPA), docosahexaenoic acid (DHA), arachidonic acid (ARA), C18:3 cis-6,9,12, and C18:3 cis-9,12,15.

### 2.2. Samples Collection and Extracts Preparation

The exhausted myrtle berries (EMB) analyzed were obtained from a local distillery. The EMB were dried at ambient temperature for a week, successively, in an air oven at 40 °C until complete drying (24 h) and stored at 0–5 °C for later uses. For analysis, seeds were manually separated from pericarps by screening after air-drying, and samples of whole EMB, seeds and pericarps were finely ground.

For polyphenolic analysis and antioxidant assays, the following extraction procedure was employed: the samples were sonicated for 60 min in a solution of 70:30 ethanol:water (*v/v*) with a sample to solvent ratio of 13:25 (*w/v*) and kept in the dark overnight. After filtration, a rotary evaporator was used to remove completely the solvent. Ultrapure water at the same volume of extraction was used to dissolve the dried samples that were then filtered using 0.20-µm syringe PVDF filters (Whatmann International Ltd., Maidstone, UK).

### 2.3. Chemical Composition

The dry matter (DM) content of the samples was determined by oven-drying at 105 °C for 24 h. The fiber fractions content (neutral detergent fiber, NDF; acid detergent fiber, ADF; and acid detergent lignin, ADL) was determined following the sequential procedure described by Van Soest, Robertson and Lewis [18], using the filter bag equipment of Ankom (Ankom Technology Corp., Fairport, NY, USA). Ash, protein (CP) and ether extract (EE) contents were determined following the analytical procedures (methods 942.05, 988.05 and 920.39, respectively) reported by AOAC [19,20]. Organic matter (% DM) was calculated as follow: 100 – ash. NFC (non-fiber carbohydrate) was calculated as follows: NFC (% DM) = 100 − (NDF + CP + ash + EE). Hemicelluloses and cellulose were calculated as NDF – ADF and ADF – ADL, respectively. Carbohydrates and gross energy were calculated according to Guimarães, Barros, Carvalho and Ferreira [21] as follow: carbohydrates (% DM) = 100 − (CP + ash + EE); and energy (kcal/100 g DM) = 4 × (CP + carbohydrate) + 9 × (EE). These parameters (except for energy) were expressed as percentage of DM. Analyses were carried out in triplicate, and results were reported as mean ± SD.

### 2.4. Fatty Acid Profile

The FA profiles of seeds, pericarps and whole EMB were determined following the method of Kramer, Fellner, Dugan, Sauer, Mossoba and Yurawecz [22] with some modifications. The powder was processed with 2 mL of 0.5 M methanolic sodium methoxide (Sigma-Aldrich, Spain) kept in a water bath at 50 °C for 10 min and then cooled at room temperature. The samples were then processed (in a water/ice bath) with 3 mL of HCl/methanol (3M) prepared, freshly, with acetyl chloride and methanol. The samples were heated again in a water bath at 50 °C for 10 min and cooled to room temperature. After adding 1 mL of solution containing methyl nonadecanoate (C19:0) as internal standard (Sigma Chemical Co., St. Louis, MO, USA) and 7.5 mL of K_2_CO_3_ (0.43 M) the samples were shaken and centrifuged (1500× *g*, room temperature, 5 min), and supernatant was kept in an amber vial for GC analysis. The Fatty acid methyl esters (FAME) determination was carried out using a 7890A GC System (Agilent Technologies, Santa Clara, CA, USA), provided with an autosampler (7693, Agilent Technologies, Santa Clara, CA, USA), a split/splitless injection port (split mode, 1:80), and a flame ionization detector (FID). FAME separation was carried out on a capillary column (CP-Sil 88, 100 m × 0.250 μm i.d., 0.25 μm film thickness, Agilent Technologies, Santa Clara, CA, USA). The oven temperature was maintained at 45 °C for 4 min, increased by 13 °C/min to 175 °C, and held for 27 min; finally, it was increased by 4 °C/min until 215 °C, and held for 35 min. Carrier gas (Helium) was used at a flow rate of 1 mL/min and with a pressure of 28 psi. Sample volume injection was 1 μL. Both injector and detector temperatures were 250 °C. Peak detection was operated using OpenLAB CDS GC ChemStation Upgrade software data system (Revision C.01.04, Agilent Technologies Inc., Santa Clara, CA, USA). Identification of individual FAME was carried out by the comparison of their retention times with those of standards methyl ester, and isomeric profiles found in the literature [23]. Analysis were carried out in triplicate, and results were expressed as mean ± SD.

### 2.5. Antioxidant Capacity

The antioxidant capacity in seeds and pericarp extracts was evaluated by two colorimetric assays measuring the activity of the samples to scavenge the two radicals DPPH (2,2-diphenyl-1-picrylhydrazyl) and ABTS (2,2′-azinobis-(3-ethylbenzothiazoline-6-sulfonic acid).

### 2.6. DPPH Radical Scavenging Activity

The DPPH radical scavenging assay was performed according to the method proposed by Brand-Williams, Cuvelier and Berset [24] with some modifications as previously reported by Maldini et al. [25]. The DPPH (2.4 mg) was dissolved in 10 mL of ethanol 70% and stored, in the dark, at −20 °C. An aliquot of 1 mL of this solution was added to 45 mL ethanol 70%, to prepare a work solution having an absorbance of 1.2 ± 0.02, at λ of 517 nm. The ethanolic extracts (100 µL) at different concentrations (from 0.1 to 100 µg) were added to the work solution, to reach 1 mL of final volume. The solutions were then mixed thoroughly and kept in the dark at 25 °C. The extent of DPPH radical reduction was measured by reading the solution’s absorbance at 517 nm at zero and after 30 min. A Trolox calibration curve in the range 0.25–7.5 μg/mL was used as positive reference. The spectrophotometer used for the assay was an Ultrospec 4300 pro UV–vis (Amersham Biosciences, Piscataway, NJ, USA), equipped with a temperature controller. Solutions were read in 1 cm quartz cuvette.

The following equation was used to calculate the scavenging activity of the DPPH radical:

% scavenging of DPPH radical = [(Ab − As)/Ab] × 100(1)

where Ab is the absorbance of the control reaction (blank); and As is the absorbance of the hydroalcoholic extracts (sample). Analyses were carried out in triplicate, and results were expressed as mean ± SD.

### 2.7. ABTS Radical Scavenging Assay

The ABTS radical scavenging assay was performed following the method detailed by Petretto et al. [26]. The assay is based on the properties of an antioxidant compound to reduce the radical cation ABTS·^+^ (chromophore blue/green) to ABTS. The extent of the reduction and the timescale depend on the concentration and the antioxidant power of the considered compound and on the duration of the reaction. The first step was the production of the radical cation obtained by the reaction between ABTS and potassium persulfate (2.45 mM) to reach a final concentration of 7 mM. The solution was kept in the dark at 25 °C for 12–16 h. Before use, the ABTS·^+^ solution was diluted with ethanol 70% to obtain a work solution with an absorbance of 0.7 ± 0.02 at λ of 734 nm. The ethanolic extracts (100 µL at different concentrations (from 0.1 to 100 µg) were added to the work solution, to reach 1 mL of final volume. The reduction of ABTS·^+^ radical cation was recorded (each concentration in triplicate) at zero and after 50 min. Antioxidant capacity of each sample was reported as percent of inhibition. In addition, the IC_50_ value (reported as mean ± SD) was calculated from regression analysis.

### 2.8. Determination of Total Phenols

Total phenols were measured by a colorimetric assay based on procedures described by Lizcano, Bakkali, Ruiz-Larrea and Ruiz-Sanz [27] with some modifications as previously described [25]. Results were expressed as µg of gallic acid equivalent (GAE) per mg of each EMB part.

### 2.9. ESI-MS and ESI-MS/MS Analysis

MS analysis was carried out using an ABSciex (Foster City, CA, USA) API4000 Q-Trap spectrometer. Depending on the investigated compound, the spectrometer was set in the both negative/positive ion mode. To optimize the experimental conditions, a solution of each standard (1 µg/mL in methanol:water 50:50) was infused at 10 µL/min into the source.

### 2.10. HPLC–ESI-MS and HPLC–ESI-MS/MS Analysis

An UHPLC system was used to perform quantitative on-line UHPLC-ESI-MS/MS analyses; the system was interfaced to an ABSciex (Foster City, CA, USA) API4000 Q-Trap instrument in Multiple Reaction Monitoring (MRM) mode, with the mass spectrometer operating as a triple quadrupole analyzer.

Liquid chromatography analysis was conducted using a Flexar UHPLC AS system (Perkin-Elmer, USA). The system was equipped with: autosampler, degasser, pump (Flexar FX-10), and column oven (PE 200). Injection volume of each sample was 5 µL and polyphenolic compounds were separated on a X Select CSH C18 column (Waters, Milford, MA, USA) (100 mm × 2.1 mm i.d., 2.5 µm d). The temperature was kept at 47 °C and 2 mobile phases were used: A (formic acid 0.1% in H_2_O) and B (formic acid 0.1% in acetonitrile). For anthocyanin compounds, a XSelect HSS T3 column (Waters, Milford, MA) (100 mm × 2.1 mm i.d., 2.5 µm d) was selected and elution was carried out at 41 °C. The flow and the solvent gradient used for elution of phenolic compounds and anthocyanins were different and were previously reported in the work of Maldini et al. [28].

For each compound, selected transitions and the optimized parameters were listed in supplementary material (Table S1). Analyst software 1.6.2. was used for the data acquisition and processing.

### 2.11. Calibration and Quantification of Phenolic Compounds

A stock solution for each standard was prepared at 1mg/mL in methanol:water (50:50). To calculate the calibration curves for each compound, five work solutions at the concentrations of 0.01, 0.05, 0.1, 1, 5 and 10 µg/mL of standards were prepared by diluting the stock solution with methanol. (each work solution was analyzed in 3 replicates).

### 2.12. Method Validation

Validation of LC–MS/MS method was performed following the guidelines of the European Medicines Agency (EMEA), concerning the analytical methods validation [29].

The determination of the limit of detection (LOD) and the limit of quantification (LOQ) for each standard compound were by the serial dilution of a stock solution until the signal:noise (S/N) ratios were 3:1 and 10:1, respectively. The LOD and LOQ values for each compound are reported in the Supplementary Materials (Table S2).

To evaluate the precision of the method, variations of intraday and interday analysis were assessed as follows: for each sample, 3 aliquots within the same day, and other 3 aliquots during three consecutive days (one per day) were analyzed. The precision of the method was expressed as percentage relative standard deviation (RSD) (Supplementary Materials, Table S2).

The efficiency of extraction and the analytical method were evaluated by performing recovery tests (in triplicate, with the optimized parameters). LC–MS/MS analysis were carried out on samples after the addition of standard solutions (at different concentration). The recovery (%) ranged from 94.6% to 106.7% within the same day.

### 2.13. Statistical Analysis

The one-way analysis of variance (ANOVA) was used to determine significance differences between seeds, pericarps and whole-EMB. Means were separated using Tukey’s test (*p* < 0.05). Differences in total phenols contents over concentrations between seeds and pericarps were assessed using linear regression in which the slope variations were compared with a global test of coincidence using an online statistical calculator (http://www.danielsoper.com/statcalc3/calc.aspx?id=103 [30]). When the data were normally distributed, the association between variables was evaluated by the Pearson product moment correlation coefficient.

## 3. Results and Discussions

The average proportions of seeds and pericarps in the whole EMB after drying at 40 °C were 58.60 ± 4.8 and 41.40 ± 4.8 (mean ± SD), respectively.

### 3.1. Chemical Composition

The results of the chemical analyses performed on whole EMB and the separated seeds and pericarps are reported in Table 1. The seeds presented slightly higher values for DM and organic matter than for pericarps (*p* < 0.01), whereas the ash content was higher for the pericarps than for seeds (*p* < 0.01). Regarding the fiber content, NDF was higher in seeds than in pericarps (*p* < 0.01), whereas ADF and lignin contents were both higher in pericarps than in seeds (*p* < 0.01). The differences in fiber content and composition for the two EMB fractions was further highlighted by the higher hemicellulose (27.75 vs. 21.24) and cellulose (25.21 vs. 8.62) contents in seeds than in pericarps (*p* < 0.01). Non-fiber carbohydrates (NFC) were more abundant in pericarps than in seeds (*p* < 0.01). The crude protein and fat contents were higher in seeds than in pericarps (*p* < 0.01); in particular, the values for crude protein and fat were about 2-fold and almost 10-fold higher in seeds compared with pericarps, respectively. These differences result in a significantly higher energy value for seeds compared with pericarps (445 vs. 384 kcal/100 g DM, *p* < 0.01). Overall, the chemical composition of whole EMB showed interesting value from a nutritional point of view, suggesting a possible use as feedstuff. This is evidenced by the value of gross energy (425 kcal/100 g of DM), which is comparable to that of typical feeds used in ruminant nutrition, as soybean meal (350–450 kcal/100 g of DM). Recently, the EMB was used as supplement in two nutritional trials in sheep [31,32], evidencing contrasting results in term of milk production (no effect or reduction of milk yield) and milk composition (no effect or reduction of protein and fat content, and reduction or no effect of milk urea content), but both studies agreed on the suitability of this by product as feed in sheep.

### 3.2. Fatty Acid Composition

The fatty acid (FA) profiles of pericarps, seeds and whole EMB are presented in Table 2. The FA profiles for EMB and seeds were very similar due to the low contribution of pericarps to the lipid content of whole EMB (see Table 1). The FA profile of pericarps showed a composition similar to that of seeds, but with a different proportion of each FA. Linoleic acid (C18:2 n-6, LA) was the most abundant FA in seeds and in whole EMB, accounting for 75% and 71% of total FA, respectively. The other most representative FAs in seeds and whole EMB were oleic acid (C18:1 cis-9, OA; 9.25% and 9.41%, respectively), palmitic acid (C16:0, PA; 8.30% and 9.34%, respectively), and stearic acid (C18:0, SA; 3.99% and 4.26%, respectively). In pericarps, the most abundant FA was PA, accounting for about 25%, followed by LA, OA, SA, arachidic acid (C20:0, AA) and LNA (17.31%, 11.69%, 8.12%, 5.20% and 4.24%, respectively). Interestingly, pericarps showed a higher proportion of LNA and saturated and unsaturated long chain FAs when compared with seeds (*p* < 0.01). In general, these results are in line with the FA composition of seeds and pericarps of fresh myrtle berries as reported in previous studies [33,34]. However, a different FA profile was reported by Cakir [35], who found the OA content of seeds and mesocarps to be 64% and 72%, respectively, with LA accounting for only 12.7% and 1.7%, respectively. This discordance could be ascribed to a difference in the maturation stage of the berries. In fact, when the variations in FA composition of myrtle berries were studied at different time points during fruit maturation [36], PA and OA were shown to be the most abundant FA in the first stage of ripening (37.03% and 21.89%, respectively), whereas their proportions decreased progressively throughout all stages of ripening (until 13.58 and 6.49%, respectively). On the other hand, the proportion of LA only accounted for 12.21% at 30 days after flowering and increased progressively to 71.34% in fully ripe fruit (180 days post flowering), thus reaching comparable values to those observed in our study.

The high amount of PUFA makes the lipid content of EMB potentially useful because of the beneficial biological and nutritional properties of these compounds. Indeed, LA could be included in cosmetic formulations since it exhibits important skin protection properties [37]. Moreover, the inclusion of lipid sources (with a high proportion of PUFA) in ruminant diets represents a useful strategy to increase the proportion of beneficial FA in meat and milk and their derived products [38]. Values of LA that exceed 50% of total FA are typical of plant oils, such as soybean, sunflower and grape seed oils [39]. In particular, the FA profile reported in our study for the myrtle seeds is very close to that of the grape seed byproduct [40], which was found to enhance the concentration of beneficial FA in sheep milk when added to the animals’ diet [14].

### 3.3. Polyphenolic Compounds

A preliminary screening of polyphenol total content was performed using the Folin–Ciocalteau method and data were expressed as µg GAE/mg of dry extract; the results are in line with those reported by Wannes and Marzouk [41] relating to fresh berry parts. As evidenced by the results (Table 3), the total polyphenol content was higher in seed extracts than pericarps (*p* < 0.01). In two recent trials on sheep nutrition, the presence of polyphenols in EMB has been associated to the reduction in blood and milk urea concentration [31] and in ammonia accumulation in rumen [42]. It seems correlated to the ability of polyphenols to bind dietary proteins and to reduce their ruminal degradation. In addition, EMB was found to be effective in reducing the proteolytic bacteria in rumen [42]. These findings also point out that *Myrtus* byproduct could be used to increase feed efficiency in animals, in terms of better protein utilization.

All secondary metabolites detected in EMB samples were identified by comparing their chromatographic behaviors and their MS and MS/MS spectra with those of standard reference compounds, when available.

The MS conditions were optimized using reference standards to achieve optimal MS sensitivity for detection and to obtain abundant fragment ions for structural elucidation. Molecules that were identified in negative ion mode belonged to the flavonoid and phenolic acid compound classes. On the other hand, due to the presence of a positive charge in the chemical structure of anthocyanin, good signal sensitivity could also be obtained in positive ion mode.

All compounds were finally confirmed by monitoring their characteristic transitions in MRM mode and comparing their retention times with those of the corresponding authentic standards.

The analytes listed in Table S1 were monitored for their occurrence and 31 compounds were identified in the investigated samples (Table 4).

The precursor/product transitions selected to develop the MRM method are described in Table S1. Quantitative results are reported in Table 4. Each of the three samples was analyzed in triplicate, and the results obtained are expressed as average values of the three analyses.

As shown, ellagic acid was found as the most representative compound in all samples with the highest content in seeds (345 mg/100 g FW), followed by whole EMB (281 mg/100 g FW) and pericarps (244 mg/100 g FW). The other most abundant acids were gallic and quinic acids, ranging 63–123 mg/100 g FW and 77–121 mg/100 g FW, respectively.

With regard to flavonoids, quercetin and quercetin 3-O-rhamnoside were the most abundant (the greatest levels being found in seeds [21 mg/100 g FW and 24 mg/100 g FW, respectively]) followed by isorhamnetin, with values in the range 8–15 mg/100 g FW. Myricetin 3-O-galactoside content was higher in pericarps (10 mg/100 g FW) than in seeds or whole EMB. Overall, the seeds contained the highest level of total polyphenols, at 566 mg/100 g FW. No anthocyanin compounds were found in our samples; this is probably because these compounds are exhaustively extracted during the hydroalcoholic infusion of the myrtle berries in liqueur production. In addition, the low stability of these compounds, which are easily degraded by light, high temperature and air, is widely reported in the literature [43].

Only few studies have assessed and quantified the polyphenolic composition of myrtle berries: three were focused on whole fresh berries [5,44,45]; one on pericarps [46]; and one specifically looked at the various myrtle berry parts [41]. Thus, a real comparison of our data with other published results is difficult. Nevertheless, the majority of secondary metabolites identified in our samples have previously been reported as present in fresh myrtle fruit; with the exception of caffeic acid, p-coumaric acid, ferulic acid, sinapic acid, quinic acid, syringic acid, chlorogenic acid, isorhamnetin, robinin, isorhamnetin 3-O-rutinoside, neohesperidin, phloridzin, apigenin, luteolin and epicatechin, which were not investigated in the cited papers.

The liquor preparation by hydroalcoholic infusion of berries, extract some of the polyphenolic compounds. Consequently, as expected, the detected levels of the main bulk of polar compounds in EMB were lower than those reported in the literature for fresh myrtle fruit, apart from ellagic acid that was more abundant in our samples. Ellagic acid is a naturally occurring phenolic compound found at high concentrations in many berries; in plants, it forms structural components in the plant cell wall and cell membrane in the form of hydrolysable tannins (ellagitannins), where it is esterified with glucose. Several papers have investigated the biological properties of ellagic acid, which include antioxidant, antimicrobial, anti-inflammatory and antimutagenic activities, as reviewed in [47].

### 3.4. Antioxidant Activity

**Figure 1:**
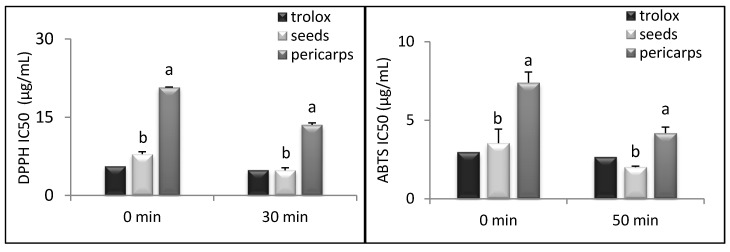
Scavenging of 50% of DPPH and ABTS radical by Trolox and ethanolic extracts from different fruit parts (seeds and pericarps) of exhausted myrtle berries (EMB) at different time points (0 and 30 min). Data were expressed as means ± SD of three independent experiments. Different letters (a,b) indicate significant differences (*p* < 0.01) between seeds and pericarps of EMB at each time point.

The free radical-scavenging properties of the exhausted myrtle berry byproduct are presented in Figure 1, where a lower IC_50_ value (µg/mL) implicates higher antioxidant activity. The ability of DPPH radical scavenging was significantly higher in seeds (*p* < 0.01) than in pericarps, with a three-fold higher antioxidant activity at both time points investigated (0 and 30 min). Our results are in line with those reported by Wannes and Marzouk [41] for the separate myrtle fruit parts, where seeds showed the highest antioxidant activity. This result could be explained by considering the higher content of phenolic acids and flavonols in seeds than in pericarps, as the antioxidant activity of fruit is mainly obtained from phenolic compounds [41].

The ABTS·^+^ assay showed that antioxidant activity was also significantly higher in seeds (*p* < 0.01) than in pericarps, with values two-fold higher at both time points (Figure 1). A highly significant positive correlation was found by comparing the results obtained using the Folin–Ciocalteau method with the DPPH and ABTS results, respectively (Table 3), confirming the well documented [48] role of phenols in antioxidant activity.

## 4. Conclusions

Our results demonstrate that exhausted myrtle berries, left over following hydroalcoholic infusion, can still provide a rich source of commercially viable phytochemicals with high antioxidant capacity, carbohydrates, proteins, lipids and polyphenols. These features and the high antioxidant activity of the byproduct support the notion that EMB, in particular the seeds, could be further processed to provide a source of bioactive compounds of bioactive compounds. The possibility of using this byproduct, in its whole form, in feed formulations should also not be excluded.

## Figures and Tables

**Table 1:** Chemical composition of seeds, pericarps and whole exhausted myrtle berries.

Chemical Composition ^1^	Pericarps	Seeds	Whole EMB	*p*-Value
dry matter (DM), %	88.58 ± 0.01 ^c^	91.37 ± 0.03 ^a^	89.73 ± 0.04 ^b^	***
organic matter, % of DM	94.46 ± 0.02 ^c^	98.05 ± 0.03 ^a^	96.53 ± 0.02 ^b^	***
NDF, % of DM	62.66 ± 1.81 ^b^	69.38 ± 0.17 ^a^	65.14 ± 1.61 ^b^	**
ADF, % of DM	59.71 ± 1.16 ^a^	51.67 ± 0.16 ^b^	53.34 ± 1.37 ^b^	***
NFC, % of DM	25.21 ± 1.83 ^a^	8.62 ± 0.17 ^c^	14.64 ± 2.05 ^b^	***
ADL, % of DM	38.47 ± 0.05 ^a^	23.92 ± 0.45 ^c^	29.85 ± 0.93 ^b^	***
hemicelluloses, % of DM	2.95 ± 0.65 ^c^	17.71 ± 0.00 ^a^	11.80 ± 0.25 ^b^	***
cellulose, % of DM	21.24 ± 1.20 ^c^	27.75 ± 0.29 ^a^	23.49 ± 0.45 ^b^	***
carbohydrates, % of DM	87.87 ± 0.03 ^a^	78.00 ± 0.16 ^c^	79.78 ± 0.48 ^b^	***
proteins, % of DM	5.35 ± 0.02 ^b^	9.43 ± 0.15 ^a^	9.02 ± 0.50 ^a^	***
fat, % of DM	1.24 ± 0.00 ^c^	10.61 ± 0.00 ^a^	7.73 ± 0.00 ^b^	***
total fatty acids, % of DM	0.39 ± 0.02 ^c^	9.17 ± 0.07 ^a^	5.92 ± 0.05 ^b^	***
ash, % of DM	5.54 ± 0.02 ^a^	1.95 ± 0.03 ^c^	3.47 ± 0.02 ^b^	***
gross energy, kcal/100 g DM	384.04 ± 0.08 ^c^	445.23 ± 0.10 ^a^	424.74 ± 0.07 ^b^	***

Means in the same row with different superscripts differ (*p* < 0.05). Values are means with standard deviation (*n* = 3). ** *p* < 0.01; *** *p* < 0.001. ^1^ NDF, neutral detergent fiber; ADF, acid detergent fiber; NFC, non-fiber carbohydrates; ADL, acid detergent lignin.

**Table 2:** Fatty acid profile (mean of FAME ± SD) of seeds, pericarps and whole exhausted barriers of myrtle resulted from liquor production.

Fatty Acids ^1^ (g/100 g of FAME)	Pericarps	Seeds	Whole EMB	*p*-Value
C10:0	1.56 ± 0.44 ^a^	0.04 ± 0.00 ^b^	0.14 ± 0.03 ^b^	***
C12:0	0.32 ± 0.06 ^a^	0.01 ± 0.00 ^b^	0.03 ± 0.00 ^b^	***
C14:0	2.47 ± 0.04 ^a^	0.05 ± 0.00 ^c^	0.21 ± 0.00 ^b^	***
C15:0	0.14 ± 0.02 ^a^	0.01 ± 0.00 ^b^	0.02 ± 0.00 ^b^	***
C16:0 (PA)	24.47 ± 0.31 ^a^	8.30 ± 0.04 ^c^	9.34 ± 0.02 ^b^	***
C16:1 *cis*-9	0.36 ± 0.05 ^a^	0.02 ± 0.00 ^b^	0.04 ± 0.00 ^b^	***
C17:0	0.00 ± 0.00 ^c^	0.11 ± 0.00 ^a^	0.11 ± 0.00 ^b^	***
C18:0 (SA)	8.12 ± 0.11 ^a^	3.99 ± 0.04 ^c^	4.26 ± 0.03 ^b^	***
C18:1 *trans*-5	0.68 ± 0.03 ^a^	0.06 ± 0.01 ^b^	0.10 ± 0.01 ^b^	***
C18:1 *trans*-6 + *trans*-8	1.03 ± 0.12 ^a^	0.11 ± 0.01 ^b^	0.17 ± 0.02 ^b^	***
C18:1 *trans*-11	0.95 ± 0.11 ^a^	0.03 ± 0.01 ^b^	0.09 ± 0.01 ^b^	***
C18:1 *cis*-9 (OA)	11.69 ± 0.12 ^a^	9.25 ± 0.06 ^b^	9.41 ± 0.05 ^b^	***
C18:1 *cis*-11	0.88 ± 0.12 ^a^	0.45 ± 0.04 ^b^	0.48 ± 0.04 ^b^	***
C18:1 *cis*-16	0.00 ± 0.00 ^b^	0.07 ± 0.00 ^a^	0.07 ± 0.00 ^a^	***
C18:2 *trans*-11,*trans*-15	1.68 ± 0.39 ^a^	0.00 ± 0.00 ^b^	0.11 ± 0.03 ^b^	***
C18:2 *n*-6 (LA)	17.31 ± 0.57 ^c^	75.09 ± 0.18 ^a^	71.38 ± 0.15 ^b^	***
C18:3 *n*-3 (LNA)	4.24 ± 0.31 ^a^	0.42 ± 0.01 ^b^	0.67 ± 0.02 ^b^	***
C20:0	5.20 ± 0.09^a^	0.58 ± 0.03 ^b^	0.88 ± 0.03 ^b^	***
C20:1 *cis*-5	0.02 ± 0.04	0.04 ± 0.01	0.04 ± 0.01	NS
C20:1 *cis*-9	0.50 ± 0.31 ^a^	0.02 ± 0.00 ^b^	0.05 ± 0.02 ^ab^	*
C20:1 *cis*-11	0.76 ± 0.25 ^a^	0.22 ± 0.00 ^b^	0.25 ± 0.02 ^b^	**
C20:2 *n*-6	0.30 ± 0.13 ^a^	0.11 ± 0.01 ^b^	0.13 ± 0.01 ^ab^	*
C20:4 *n*-6	1.32 ± 0.26 ^a^	0.03 ± 0.00 ^b^	0.11 ± 0.02 ^b^	***
C22:0	4.03 ± 0.13 ^a^	0.11 ± 0.00 ^c^	0.36 ± 0.01 ^b^	***
C22:3 *n*-6	0.33 ± 0.05 ^a^	0.14 ± 0.00 ^b^	0.15 ± 0.00 ^b^	***
EPA	0.00 ± 0.00 ^b^	0.02 ± 0.01 ^a^	0.02 ± 0.01 ^a^	*
C24:0	3.77 ± 0.15 ^a^	0.10 ± 0.01 ^c^	0.34 ± 0.01 ^b^	***
C24:1 *cis*-15	0.43 ± 0.00 ^a^	0.20 ± 0.00 ^c^	0.21 ± 0.00 ^b^	***
C25:0	0.80 ± 0.09 ^a^	0.02 ± 0.00 ^b^	0.07 ± 0.01 ^b^	***
C26:0	3.61 ± 0.63^a^	0.02 ± 0.00 ^b^	0.25 ± 0.04 ^b^	***
Σ SFA	55.04 ± 0.97 ^a^	13.41 ± 0.09 ^c^	16.09 ± 0.02 ^b^	***
Σ MUFA	17.31 ± 0.39 ^a^	10.47 ± 0.07 ^b^	10.91 ± 0.09 ^b^	***
Σ PUFA	25.28 ± 1.13 ^c^	76.05 ± 0.14 ^a^	72.79 ± 0.12 ^b^	***
SFA:PUFA ratio	2.18 ± 0.13 ^a^	0.18 ± 0.00 ^b^	0.22 ± 0.00 ^b^	***
Σ C18 1 *trans*	2.66 ± 0.22 ^a^	0.20 ± 0.02 ^b^	0.36 ± 0.02 ^b^	***
Σ C18:2 (except LA)	1.77 ± 0.55 ^a^	0.22 ± 0.05 ^b^	0.32 ± 0.03 ^b^	**
Σ C21:1	1.28 ± 0.07 ^a^	0.28 ± 0.01 ^b^	0.35 ± 0.01 ^b^	**

Means in the same row with different superscripts differ (*p* < 0.05). NS *p* > 0.05; * *p* < 0.05; ** *p* < 0.01; *** *p* < 0.001. ^1^ FAME, fatty acid methyl ester; PA, palmitic acid; SA, stearic acid; OA, oleic acid; LA, linoleic acid; LNA, α-linolenic acid; EPA, eicosapentaenoic acid; SFA, total saturated fatty acids; MUFA, total monounsaturated fatty acids; PUFA, total polyunsaturated fatty acids.

**Table 3:** Determination of total phenols by the Folin–Ciocalteau method in extracted of seeds and pericarps of EMB. Data are expressed as mean ± SD of 3 independent experiments. Each result showed a positive correlation (*p* < 0.001) with DPPH and ABTS results.

Extract Concentration (µg/µL)	Seeds (µg GAE ^1^)	Pericarps (µg GAE)	Correlation with Antioxidant Activity *p*-Value
100	468.96 ± 2.95	158.99 ± 11.95	*p* < 0.001
50	248.85 ± 2.59	82.19 ± 6.81	
25	136.56 ± 9.61	31.02 ± 2.09	
10	50.39 ± 1.62	19.70 ± 3.62	
5	30.99 ± 1.32	8.63 ± 0.60	
1	7.22 ± 0.57	1.60 ± 0.55	

^1^ GAE, gallic acid equivalent.

**Table 4:** Polyphenolic contents (mg/100 g DW ± standard deviation) and percentages (%) of different part of exhausted berries of *Myrtus communis*.

Compound	tR	Pericarps		Seeds		Whole EMB		*p*-Value
			mg/100 g ± SD	%	mg/100 g ± SD	%	mg/100 g ± SD		%
gallic acid	3.27	78.49 ± 5.63	15.02	63.44 ± 4.52	11.22	65.50 ± 9.95	12.70	NS
caffeic acid	9.27	0.07 ± 0.01	0.01	0.06 ± 0.01	0.01	0.07 ± 0.01	0.01	NS
*p* coumaric acid	10.26	0.51 ± 0.02 ^a^	0.10	0.17 ± 0.01	0.03	0.27 ± 0.01 ^ab^	0.05	**
ellagic acid	11.42	244.67 ± 14.63 ^c^	46.83	345.02 ± 5.95 ^a^	61.00	281.79 ± 19.16 ^b^	54.64	***
ferulic acid	11.38	0.15 ± 0.00 ^b^	0.03	0.20 ± 0.02 ^a^	0.04	0.18 ± 0.01 ^a^	0.04	***
sinapic acid	11.47	0.02 ± 0.01 ^c^	0.00	0.04 ± 0.01 ^a^	0.01	0.03 ± 0.01 ^b^	0.01	***
quinic acid	1.30	120.82 ± 3.65 ^a^	23.13	77.11 ± 1.44 ^c^	13.63	96.36 ± 0.60 ^b^	18.68	***
siringic acid	9.81	7.48 ± 0.81 ^a^	1.43	0.51 ± 0.06 ^b^	0.09	2.77 ± 0.08 ^ab^	0.54	**
chlorogenic acid	8.93	0.09 ± 0.01	0.02	0.08 ± 0.01	0.01	0.08 ± 0.01	0.02	NS
kaempferol	16.11	1.81 ± 0.04 ^b^	0.35	2.07 ± 0.04 ^a^	0.37	1.80 ± 0.15 ^b^	0.35	*
quercetin	15.42	18.76 ± 0.45 ^b^	3.59	20.91 ± 1.01 ^a^	3.70	19.18 ± 0.31 ^b^	3.72	*
isorhamnetin	16.19	7.67 ± 0.51 ^c^	1.47	14.75 ± 0.37 ^a^	2.61	9.83 ± 0.38 ^b^	1.91	***
myricetin	12.64	7.11 ± 0.31 ^a^	1.36	5.26 ± 0.30 ^b^	0.93	5.87 ± 0.56 ^b^	1.14	**
isoquercetin	11.63	2.30 ± 0.08 ^a^	0.44	2.21 ± 0.06 ^a^	0.39	1.99 ± 0.05 ^b^	0.39	**
quercetin 3-*O*-rhamnoside	12.74	17.24 ± 0.52 ^c^	3.30	23.78 ± 0.43 ^a^	4.20	19.15 ± 0.26 ^b^	3.71	***
robinin	10.77	0.03 ± 0.01 ^a^	0.01	0.02 ± 0.01 ^b^	0.00	0.02 ± 0.01 ^b^	0.00	**
rutin	11.43	0.02 ± 0.01 ^ab^	0.00	0.01 ± 0.01 ^b^	0.00	0.03 ± 0.01 ^a^	0.01	*
isorhamnetin 3-*O*-rutinoside	12.41	0.00 ± 0.00 ^a^	0.00	0.00 ± 0.00 ^c^	0.00	0.00 ± 0.00 ^b^	0.00	***
quercetin 3-*O*-galactoside	11.71	0.32 ± 0.01 ^a^	0.06	0.13 ± 0.01 ^c^	0.02	0.18 ± 0.01 ^b^	0.04	***
myricitrin	11.46	4.62 ± 0.10 ^c^	0.88	6.80 ± 0.16 ^a^	1.20	5.26 ± 0.28 ^b^	1.02	***
neohesperidin	13.11	0.02 ± 0.01 ^c^	0.00	0.05 ± 0.01 ^a^	0.01	0.03 ± 0.01 ^b^	0.00	***
myricetin 3-*O*-galactoside	10.60	9.55 ± 0.06 ^a^	1.83	2.65 ± 0.08 ^c^	0.47	4.91 ± 0.20 ^b^	0.95	***
phloridzin	13.19	0.05 ± 0.01 ^a^	0.01	0.04 ± 0.01 ^b^	0.01	0.04 ± 0.01 ^b^	0.01	***
apigenin	16.03	0.01 ± 0.00 ^a^	0.00	0.00 ± 0.00 ^b^	0.00	0.00 ± 0.00 ^b^	0.00	**
luteolin	15.38	0.01 ± 0.00	0.00	0.01 ± 0.00	0.00	0.01 ± 0.00	0.00	NS
epicatechin	8.72	0.06 ± 0.01 ^a^	0.01	0.04 ± 0.01 ^b^	0.01	0.04 ± 0.01 ^b^	0.01	***
catechin	9.61	0.11 ± 0.01 ^a^	0.02	0.09 ± 0.01 ^b^	0.02	0.08 ± 0.01 ^c^	0.02	***
epigallocatechin	7.68	0.05 ± 0.01 ^a^	0.01	0.03 ± 0.01 ^b^	0.01	0.04 ± 0.01 ^b^	0.01	**
epigallocatechin 3-*O*-gallate	9.28	0.40 ± 0.03 ^a^	0.08	0.13 ± 0.01 ^c^	0.02	0.21 ± 0.01 ^b^	0.04	***
procyanidin B1	7.96	0.02 ± 0.01 ^a^	0.00	0.00 ± 0.00 ^c^	0.00	0.02 ± 0.01 ^b^	0.00	***
procyanidin B2	8.82	0.02 ± 0.01 ^a^	0.00	0.00 ± 0.00 ^b^	0.00	0.02 ± 0.01 ^ab^	0.00	**
cyanidin 3,5-di-*O*-glucoside	9.65	ND		ND		ND		
cyanidin 3-*O*-glucoside	10.50	ND		ND		ND		
cyanidin 3-*O*-arabinoside	11.86	ND		ND		ND		
delphinidin 3-*O*-glucoside	10.00	ND		ND		ND		
malvidin 3-*O*-glucoside	12.01	ND		ND		ND		
pelargonidin 3-*O*-glucoside	13.94	ND		ND		ND		
pelargonidin 3-*O*-rutinoside	14.41	ND		ND		ND		

Means in the same row with different superscripts differ (*p* < 0.05). NS *p* > 0.05; * *p* < 0.05; ** *p* < 0.01; *** *p* < 0.001. ND, not determined (below LOD).

## Supplementary Materials

The following are available online at https://www.mdpi.com/2304-8158/8/7/237/s1, Table S1: LC–MS/MS conditions for quantification of detected compounds by negative/positive ion MRM, Table S2: Accuracy, precision, linearity, LOQ and LOD of LC-ESI-QqQ-MS/MS MRM method for the analysis of standard compounds.

## References

[1] Federici F., Fava F., Kalogerakis N., Mantzavinos D. (2009). Valorisation of agro-industrial by-products, effluents and waste: Concept, opportunities and the case of olive mill wastewaters. J. Chem. Technol. Biotechnol..

[2] Mauricio E.M., Rosado C., Duarte M.P., Fernando A.L., Diaz-Lanza A.M. (2018). Evaluation of Industrial Sour Cherry Liquor Wastes as an Ecofriendly Source of Added Value Chemical Compounds and Energy. Waste Biomass Valorization.

[3] Balasundram N., Sundram K., Samman S. (2006). Phenolic compounds in plants and agri-industrial by-products: Antioxidant activity, occurrence, and potential uses. Food Chem..

[4] Quideau S., Deffieux D., Douat-Casassus C., Pouysegu L. (2011). Plant polyphenols: Chemical properties, biological activities, and synthesis. Angew. Chem. Int. Ed..

[5] Tuberoso C.I.G., Rosa A., Bifulco E., Melis M.P., Atzeri A., Pirisi F.M., Dessì M.A. (2010). Chemical composition and antioxidant activities of *Myrtus communis* L. berries extracts. Food Chem..

[6] Quiñones M., Miguel M., Aleixandre A. (2013). Beneficial effects of polyphenols on cardiovascular disease. Pharmacol. Res..

[7] Saito M., Sakagami H., Fujisawa S. (2002). Cytotoxicity and apoptosis induction by butylated hydroxyanisole (BHA) and butylated hydroxytoluene (BHT). Anticancer Res..

[8] Anunciato T.P., da Rocha Filho P.A. (2012). Carotenoids and polyphenols in nutricosmetics, nutraceuticals, and cosmeceuticals. J. Cosmet. Dermatol..

[9] Asensi M., Ortega A., Mena S., Feddi F., Estrela J.M. (2011). Natural polyphenols in cancer therapy. Crit. Rev. Clin. Lab. Sci..

[10] Aerts R.J., Barry T.N., McNabb W.C. (1999). Polyphenols and agriculture: Beneficial effects of proanthocyanidins in forages. Agric. Ecosyst. Environ..

[11] Grainger C., Clarke T., Auldist M., Beauchemin K., McGinn S., Waghorn G., Eckard R. (2009). Potential use of Acacia mearnsii condensed tannins to reduce methane emissions and nitrogen excretion from grazing dairy cows. Can. J. Anim. Sci..

[12] Vasta V., Yanez-Ruiz D.R., Mele M., Serra A., Luciano G., Lanza M., Biondi L., Priolo A. (2010). Bacterial and protozoal communities and fatty acid profile in the rumen of sheep fed a diet containing added tannins. Appl. Environ. Microb..

[13] Nudda A., Correddu F., Marzano A., Battacone G., Nicolussi P., Bonelli P., Pulina G. (2015). Effects of diets containing grape seed, linseed or both on milk production traits, liver and kidney activities, and immunity of lactating dairy ewes. J. Dairy Sci..

[14] Correddu F., Gaspa G., Pulina G., Nudda A. (2016). Grape seed and linseed, alone and in combination, enhance unsaturated fatty acids in the milk of Sarda dairy sheep. J. Dairy Sci..

[15] Dung N.T., Van Binh D., Mui N.T., Preston T. (2010). Effect of cassava hay supplementation on milk production in lactating goats. Livest. Res. Rural Dev..

[16] Franco M.A., Versini G., Saba R. (1998). Caratterizzazione del Liquore “Mirto di Sardegna Tradizionale”.

[17] Tuberoso C.I.G., Barra A., Cabras P. (2008). Effect of different technological processes on the chemical composition of myrtle (*Myrtus communis* L.) alcoholic extracts. Eur. Food Res. Technol..

[18] Van Soest P.J., Robertson J.B., Lewis B.A. (1991). Methods for dietary fiber, neutral detergent fiber, and nonstarch polysaccharides in relation to animal nutrition. J. Dairy Sci..

[19] AOAC (2000). Official Methods of Analysis of AOAC International.

[20] AOAC (2005). Official Methods of Analysis of AOAC International.

[21] Guimarães R., Barros L., Carvalho A.M., Ferreira I.C.F.R. (2010). Studies on chemical constituents and bioactivity of rosa micrantha: An alternative antioxidants source for food, pharmaceutical, or cosmetic applications. J. Agric. Food Chem..

[22] Kramer J.K.G., Fellner V., Dugan M.E.R., Sauer F.D., Mossoba M.M., Yurawecz M.P. (1997). Evaluating acid and base catalysts in the methylation of milk and rumen fatty acids with special emphasis on conjugated dienes and total trans fatty acids. Lipids.

[23] Kramer J.K., Cruz-Hernandez C., Deng Z., Zhou J., Jahreis G., Dugan M.E. (2004). Analysis of conjugated linoleic acid and trans 18: 1 isomers in synthetic and animal products. Am. J. Clin. Nutr..

[24] Brand-Williams W., Cuvelier M.E., Berset C. (1995). Use of free radical method to evaluate antioxidant activity. LWT-Food Sci. Technol..

[25] Maldini M., Montoro P., Addis R., Toniolo C., Petretto G.L., Foddai M., Nicoletti M., Pintore G. (2016). A new approach to discriminate Rosmarinus officinalis L. plants with antioxidant activity, based on HPTLC fingerprint and targeted phenolic analysis combined with PCA. Ind. Crops Prod..

[26] Petretto G.L., Maldini M., Addis R., Chessa M., Foddai M., Rourke J.P., Pintore G. (2016). Variability of chemical composition and antioxidant activity of essential oils between *Myrtus communis* var. Leucocarpa DC and var. Melanocarpa DC. Food Chem..

[27] Lizcano L.J., Bakkali F., Ruiz-Larrea M.B., Ruiz-Sanz J.I. (2010). Antioxidant activity and polyphenol content of aqueous extracts from Colombian Amazonian plants with medicinal use. Food Chem..

[28] Maldini M., Foddai M., Natella F., Addis R., Chessa M., Petretto G.L., Tuberoso C.I.G., Pintore G. (2016). Metabolomic study of wild and cultivated caper (Capparis spinosa L.) from different areas of Sardinia and their comparative evaluation. J. Mass Spectrom..

[29] EMEA, European Medicines Agency (1995). Quality Guidelines: Validation of Analytical Procedures: Text and Methodology (ICHQ2). http://www.ema.europa.eu/ema/index.jsp?curl=pages/regulation/general/general_content_000768.jsp&mid=WC0b01ac0580028e8d/.

[30] Free Statistics Calculators. http://www.danielsoper.com/statcalc3/calc.aspx?id=103.

[31] Nudda A., Correddu F., Atzori A.S., Marzano A., Battacone G., Nicolussi P., Bonelli P., Pulina G. (2017). Whole exhausted berries of *Myrtus communis* L. supplied to dairy ewes: Effects on milk production traits and blood metabolites. Small Rumin. Res..

[32] Nudda A., Buffa G., Atzori A.S., Cappai M.G., Caboni P., Fais G., Pulina G. (2019). Small amounts of agro-industrial byproducts in dairy ewes diets affects milk production traits and hematological parameters. Anim. Feed Sci. Technol..

[33] Wannes W.A., Marzouk B. (2016). Characterization of myrtle seed (*Myrtus communis* var. baetica) as a source of lipids, phenolics, and antioxidant activities. J. Food Drug Anal..

[34] Wannes W.A., Mhamdi B., Sriti J., Bettaieb I., Tounsi M.S., Marzouk B. (2011). Fatty acid and glycerolipid changes during Tunisian myrtle (*Myrtus communis* var. italica) fruit ripening. J. Food Biochem..

[35] Cakir A. (2004). Essential oil and fatty acid composition of the fruits of Hippophae rhamnoides L. (Sea Buckthorn) and *Myrtus communis* L. from Turkey. Biochem. Syst. Ecol..

[36] Wannes W.A., Mhamdi B., Marzouk B. (2009). Variations in essential oil and fatty acid composition during *Myrtus communis* var. italica fruit maturation. Food Chem..

[37] Nguyen M.T., Hanzelmann D., Härtner T., Peschel A., Götz F. (2016). Skin-Specific unsaturated fatty acids boost the Staphylococcus aureus innate immune response. Infect. Immun..

[38] Shingfield K.J., Bonnet M., Scollan N.D. (2013). Recent developments in altering the fatty acid composition of ruminant-derived foods. Animal.

[39] Ramos M.J., Fernández C.M., Casas A., Rodríguez L., Pérez Á. (2009). Influence of fatty acid composition of raw materials on biodiesel properties. Bioresour. Technol..

[40] Correddu F., Nudda A., Battacone G., Boe R., Francesconi A.H.D., Pulina G. (2015). Effects of grape seed supplementation, alone or associated with linseed, on ruminal metabolism in Sarda dairy sheep. Anim. Feed Sci. Technol..

[41] Wannes W.A., Marzouk B. (2013). Differences between myrtle fruit parts (*Myrtus communis* var. italica) in phenolics and antioxidant contents. J. Food Biochem..

[42] Correddu F., Fancello F., Chessa L., Atzori A.S., Pulina G., Nudda A. (2019). Effects of supplementation with exhausted myrtle berries on rumen function of dairy sheep. Small Rumin. Res..

[43] Montoro P., Tuberoso C.I., Piacente S., Perrone A., De Feo V., Cabras P., Pizza C. (2006). Stability and antioxidant activity of polyphenols in extracts of *Myrtus communis* L. berries used for the preparation of myrtle liqueur. J. Pharm. Biomed. Anal..

[44] Barboni T., Cannac M., Massi L., Perez-Ramirez Y., Chiaramonti N. (2010). Variability of polyphenol compounds in *Myrtus communis* L. (Myrtaceae) berries from Corsica. Molecules.

[45] Barboni T., Venturini N., Paolini J., Desjobert J.M., Chiaramonti N., Costa J. (2010). Characterisation of volatiles and polyphenols for quality assessment of alcoholic beverages prepared from Corsican *Myrtus communis* berries. Food Chem..

[46] Martín T., Rubio B., Villaescusa L., Fernández L., Díaz A.M. (1999). Polyphenolic compounds from pericarps of *Myrtus communis*. Pharm. Biol..

[47] Vattem D.A., Shetty K. (2005). Biological functionality of ellagic acid: A review. J. Food Biochem..

[48] Petretto G.L., Tuberoso C.I.G., Vlahopoulou G., Atzei A., Mannu A., Zrira S., Pintore G. (2016). Volatiles, color characteristics and other physico-chemical parameters of commercial Moroccan honeys. Nat. Prod. Res..

