# Supplementary material for: Myrtus Communis Liquor Byproduct as a Source of Bioactive Compounds
Source: Foods. 2019 Jun 30;8(7):237. doi: 10.3390/foods8070237 (PMC6678195; doi:10.3390/foods8070237)
Supplement: Supplementary file 1 [file foods-08-00237-s001.pdf]

Supplementary material

Table S1. LC–MS/MS conditions for quantification of detected compounds by negative/positive ion MRM

Compound	Selected transitions	Dwell (msec)	DP	EP	CE	CXP
isoquercetin	463/299.9	20	-115	-3.76	-40	-17.5
quercetin 3-*O*-rhamnoside	447/300	20	-97.15	-14	-34	-18
phloridzin	435/273	20	-89	-3.9	-22.2	-47.8
robinin	739/593	20	-126	-10.7	-42.6	-32.2
rutin	609/300	20	-125	-10.5	-60	-13.6
isorhamnetin 3-*O*-rutinoside	623/315	20	-90.8	-10.8	-46.7	-17.7
quercetin 3-*O*-galactoside	463/300	20	-104.7	-10.6	-40.2	-15
myricitrin	463/316	20	-97.8	-10.7	-40.6	-17.7
myricetin 3-*O*-galactoside	479/316	20	-95.6	-15	-37.7	-16.1
neohesperidin	609/301	20	-94	-15	-38.8	-14.2
luteolin	285/133	20	-97.6	-10.7	-50.6	-16.6
kaempferol	285/186.9	20	-113	-8.7	-39.2	-9.7
quercetin	301/151	20	-94.6	-15	-30.6	-7
isorhamnetin	315/300	20	-111.4	-15	-31.9	-17.9
myricetin	317/151	20	-60	-10	-40	-15
apigenin	269/117	20	-107.8	-15	-45.1	-4.7
ellagic acid	301/283	20	-101	-13	-44.7	-15.2
epicatechin	289/245	20	-93	-9	-21.7	-14.6
catechin	289/245	20	-93	-9	-21.7	-14.6
epigallocatechin	305/125	20	-87.3	-10.7	-29	-4.9
epigallocatechin gallate	457/169	20	-89	-10.8	-22.1	-8.2
procyanidin B1	577/289	20	-99.8	-3.5	-22.3	-7.9
procyanidin B2	577/289	20	-99.2	-3	-35	-17.7
chlorogenic acid	353/191	20	-71.1	-5.1	-21.1	-10.7
p-coumaric acid	163/119	20	-56.3	-2	-20.04	-8
ferulic acid	193/134	20	-58.2	-11	-20	-5.8
sinapic acid	223/164	20	-53.9	-11	-18.3	-8.2
gallic acid	169/125	20	-58.9	-6	-19.7	-4.9
caffeic acid	179/135	20	-69.6	-13.9	-19.1	-23
quinic acid	191/85	20	-84.1	-15	-32	-13.9
syringic acid	197/121	20	-52	-10.7	-24.8	-4.5
cyanidin 3,5-di-*O*-glucoside	611/287	100	177	14	55	19
cyanidin 3-*O*-glucoside	449/287	100	192	12	27.2	20.5
cyanidin 3-*O*-arabinoside	419/287	100	194	11.5	24	19.7
delphinidin 3-*O*-glucoside	465/303	100	181	14	30	21

malvidin 3-*O*-glucoside	493/331	100	94	12	29	21
pelargonidin 3-*O*-glucoside	433/271	100	76.9	10.1	28.8	18.8
pelargonidin 3-*O*-rutinoside	579/271	100	98	13	40	18.4

DP= declustering potential; EP=entrance potential; CE= collision energy; CXP = cell exit potential.

Table S2. Accuracy, precision, linearity, LOQ and LOD of LC-ESI-QqQ-MS/MS MRM method for the analysis of standard compounds.

Compound	Calibration curve	r^2	Intra-day (CV%)	Inter-day (CV%)	LOQ (μg/mL)	LOD (μg/mL)
isoquercetin	$y=7.05^{e5}x-5.69^{e3}$	0.999	1.03	3.56	0.008	0.003
quercetin 3-*O*-rhamnoside	$y=2.1^{e6}x-1.4^{e4}$	0.999	1.99	2.52	0.006	0.002
phloridzin	$y=2.14^{e6}x+1.22^{e3}$	0.999	2.96	3.36	0.008	0.003
robinin	$y=1.29^{e6}x-1.28^{e4}$	0.999	3.55	6.73	0.012	0.004
rutin	$y=8.09^{e5}x+1.08^{e3}$	0.999	4.24	5.03	0.009	0.003
isorhamnetin 3-*O*-rutinoside	$y=1.81^{e6}x+1.17^{e3}$	0.999	4.64	7.61	0.007	0.002
quercetin 3-*O*-galactoside	$y=2.74^{e6}x+1.32^{e4}$	0.999	1.71	2.85	0.005	0.001
myricitrin	$y=1.78^{e6}x-7.27^{e3}$	0.999	5.14	6.03	0.004	0.001
myricetin 3-*O*-galactoside	$y=1.6^{e6}x-1.54^{e4}$	0.998	2.57	4.35	0.008	0.002
neohesperidin	$y=1.79^{e6}x+2.95^{e3}$	0.999	1.55	3.76	0.006	0.002
luteolin	$y=1.85^{e6}x-9.48^{e3}$	0.999	3.99	4.48	0.005	0.001
kaempferol	$y=9.95^{e4}x-935$	0.999	2.59	4.22	0.009	0.004
quercetin	$y=3.91^{e5}x+2.28^{e4}$	0.999	4.39	6.47	0.041	0.013
isorhamnetin	$y=2.52^{e6}x+1.16^{e4}$	0.998	5.23	7.09	0.007	0.003
myricetin	$y=2.33^{e5}x-396$	0.999	0.58	2.16	0.006	0.002
apigenin	$y=1.56^{e6}x-1.75^{e3}$	0.997	1.91	3.22	0.002	0.001
ellagic acid	$y=2.35^{e4}x-2.04^{e4}$	0.993	6.34	7.33	0.085	0.026
epicatechin	$y=6.95^{e5}x-8.29^{e3}$	0.999	5.65	7.12	0.009	0.003
catechin	$y=6.42^{e5}x-352$	0.999	3.87	5.52	0.004	0.001
epigallocatechin	$y=7.83^{e5}x-7.68^{e3}$	0.999	7.93	8.72	0.009	0.003
epigallocatechin gallate	$y=2.45^{e6}x-1.96^{e4}$	0.998	8.29	9.33	0.008	0.002
procyanidin B1	$y=7.5^{e5}x-5.69^{e3}$	0.999	6.67	8.05	0.008	0.002
procyanidin B2	$y=7.26^{e5}x-6.69^{e3}$	0.999	1.27	3.23	0.009	0.003
chlorogenic acid	$y=1.71^{e6}x-5.28^{e4}$	0.998	4.19	5.67	0.035	0.011
p-coumaric acid	$y=3.69^{e6}x+2.38^{e4}$	0.999	1.24	4.55	0.008	0.002
ferulic acid	$y=1.28^{e6}x-3.63^{e3}$	0.999	4.53	5.03	0.008	0.003
sinapic acid	$y=3.85^{e5}x-2.12^{e3}$	0.997	1.47	2.87	0.007	0.002
gallic acid	$y=2.35^{e5}x-8.33^{e3}$	0.999	0.61	2.11	0.021	0.006
caffeic acid	$y=2.31^{e6}x+4.55^{e3}$	0.998	1.24	3.15	0.042	0.013
quinic acid	$y=3.75^{e5}x-3.65^{e3}$	0.999	7.65	8.95	0.011	0.003
syringic acid	$y=6.64^{e4}x+350$	0.999	3.85	5.25	0.027	0.008

cyanidin 3,5-di-*O*-glucoside	$y=5,98^{e5}x+4,31^{e5}$	0.993	1.97	4.06	0.008	0.002
cyanidin 3-*O*-glucoside	$y=6,14^{e4}x-2,03^{e4}$	0.994	3.42	6.72	0.010	0.003
cyanidin 3-*O*-arabinoside	$y=1,5^{e6}x+4,38^{e6}$	0.993	0.76	4.24	0.090	0.020
delphinidin 3-*O*-glucoside	$y=2,01^{e6}x+5,46^{e4}$	0.998	1.59	2.95	0.050	0.012
malvidin 3-*O*-glucoside	$y=1,56^{e6}x+3,29^{e6}$	0.994	4.31	5.56	0.007	0.0003
pelargonidin 3-*O*-glucoside	$y=1.53^{e7}x+4.4^{e4}$	0.999	4.5	6.01	0.004	0.001
pelargonidin 3-*O*-rutinoside	$y=7.47^{e6}x-1.26^{e4}$	0.999	4.95	5.48	0.007	0.003
